# Antioxidant Activities, Total Phenolics and Flavonoids Content in Two Varieties of Malaysia Young Ginger (*Zingiber officinale *Roscoe)

**DOI:** 10.3390/molecules15064324

**Published:** 2010-06-14

**Authors:** Ali Ghasemzadeh, Hawa Z. E. Jaafar, Asmah Rahmat

**Affiliations:** 1 Department of Crop Science, Faculty of Agriculture, University Putra Malaysia, 43400 UPM Serdang, Selangor, Malaysia; E-Mail: upmali@yahoo.com (A.G.); 2 Department of Nutrition & Dietetics, Faculty of Medicine & Health Sciences, University Putra Malaysia, 43400 UPM Serdang, Selangor, Malaysia

**Keywords:** *Zingiber officinale*, DPPH, FRAP, total phenolics, total flavonoids

## Abstract

Ginger (*Zingiber officinale *Roscoe) is a well known and widely used herb, especially in Asia, which contains several interesting bioactive constituents and possesses health promoting properties. In this study, the antioxidant activities of methanol extracts from the leaves, stems and rhizomes of two *Zingiber officinale *varieties (Halia Bentong and Halia Bara) were assessed in an effort to compare and validate the medicinal potential of the subterranean part of the young ginger. The antioxidant activity and phenolic contents of the leaves as determined by the 1,1-diphenyl-2-picryl-hydrazyl (DPPH) assay and the total amounts of phenolics and flavonoids were higher than those of the rhizomes and stems. On the other hand, the ferric reducing/antioxidant potential (FRAP) activity of the rhizomes was higher than that of the leaves. At low concentration the values of the leaves’ inhibition activity in both varieties were significantly higher than or comparable to those of the young rhizomes. Halia Bara had higher antioxidant activities as well as total contents of phenolic and flavonoid in comparison with Halia Bentong. This study validated the medicinal potential of the leaves and young rhizome of *Zingiber officinale *(Halia Bara) and the positive relationship between total phenolics content and antioxidant activities in *Zingiber officinale.*

## 1. Introduction

Plants such as herbs have long been used in traditional/folk medicine in various cultures throughout the world. *Zingiber officinale* is one of these traditional folk medicinal plants that have been used for over 2000 years by Polynesians for treating diabetes, high blood pressure, cancer, fitness and many other illnesses [1]. 

Generation of free radicals or reactive oxygen species (ROS) during metabolism and other activities beyond the antioxidant capacity of a biological system gives rise to oxidative stress [2], which plays a role in heart diseases, neurodegenerative diseases, cancer and in the aging process [3]. This concept is supported by increasing evidence indicating that oxidative damage plays a role in the development of chronic, age-related degenerative diseases, and that dietary antioxidants oppose this, thus lowering the risk of disease [4]. Antioxidants are substances that when present in low concentrations, compared to those of an oxidisable substrate significantly delay or prevent oxidation of that substance [5]. 

Apart from their role as health benefactors, antioxidants are also added to food to prevent or delay its oxidation, normally initiated by free radicals formed during the food’s exposure to environmental factors such as air, light and temperature [6]. At present most of the antioxidants used for this are manufactured synthetically. The main disadvantage with the synthetic antioxidants is their side effects when taken *in vivo* [7]. Strict governmental rules regarding the safety of the food has necessitated the search for alternatives as food preservatives [8]. 

Plants are a potential source of natural antioxidants. Natural antioxidants or phytochemical antioxidants are secondary metabolites of plants [9]. Carotenoids, flavonoids, cinnamic acids, benzoic acids, folic acid, ascorbic acid, tocopherols, tocotrienols, *etc*. are among the antioxidants produced by plants for their own sustenance. Beta-carotene, ascorbic acid and alpha tocopherols are widely used antioxidants [10]. *Zingiber officinale* contains a number of antioxidants such as beta-carotene, ascorbic acid, terpenoids, alkaloids, and polyphenols such as flavonoids, flavones glycosides, rutin, *etc.* [11]. Easily cultivable, *Zingiber officinale* with its wide range of antioxidants can be a major source of natural or phytochemical antioxidants [12]. Although various extracts are obtained from ginger, it is the CO_2_ extracts that are richest in polyphenol compounds and have a composition that closely resembles that of the rhizomes [13,14]. The method of preparation has been used in commercial ginger preparation, since ginger has been widely speculated to be beneficial for human health because it exerts antioxidant activity [11]. Previous studies on the antioxidant properties of various ginger species had been confined only to the rhizomes [8,15], which have been reported to have tyrosinase inhibiting properties [16]. Recently, skin-lightening cosmeceutical products have been developed from the rhizomes of gingers [17]. Although the leaves of ginger species have been used for food flavouring and in traditional medicine, insufficient research has been done on their antioxidant and tyrosinase inhibiting properties. 

Antioxidants affect the process of lipid oxidation at different stages due to the differences in their mode of action. Because of the complexity of the oxidation process itself, the diversity of the substrates and the active species involved, the application of different test methods is necessary to evaluation antioxidants. The objectives of the present study are to determine the antioxidant activity, the total phenolic content, and the total flavonoid content of methanolic extracts from two varieties of Malaysian Gingers (*Zingiber officinale), *namely Halia Bentong and Halia Bara.

## 2. Results and Discussion

### 2.1. Total phenolics (TP) and total flavonoids (TF)

The level of phenolic compounds in methanolic extracts of the leaves, rhizomes and stems in the two varieties of *Zingiber officinale* are presented in Table 1. Polyphenolic compounds are known to have antioxidant activity and it is likely that the activity of the extracts is due to these compounds [10,18,19,20,21,22]. This activity is believed to be mainly due to their redox properties, which plays an important role in adsorbing and neutralizing free radicals, quenching singlet and triplet oxygen, or decomposing peroxides [15,23].

**Table 1:** Total phenolic and flavonoid contents of the methanolic extracts in different parts of two varieties of *Zingiber officinale*.

	Variety	Leave	Stem	Rhizome
**Total Flavonoids ^a^**	Halia Bentong	5.54 ± 1.83	1.36 ± 0.85	3.66 ± 0.45
	Halia Bara	7.05 ± 7.4	1.77 ± 0.75	4.21 ± 0.98
**Total phenolics ^b^**	Halia Bentong	33.0 ± 1.13	7.8 ± 0.65	10.22 ± 0.87
	Halia Bara	39.1 ± 9.2	8.5 ± 0.81	13.5 ± 2.26

All analyses are the mean of triplicate measurements ± standard deviation; a: Expressed as mg quercetin/g of dry plant material; b: Expressed as mg gallic acid/g of dry plant material.

In both varieties, the total flavonoid and phenolic contents in the leaves were more than in the rhizomes, followed by contents in the stems. Comparing the varieties, it was found that Halia Bara had higher contents of TP (16.3%) and TF (20%) than Halia Bentong. Differences between the varieties and also the parts of plants were highly significant (p ≤ 0.001). The total content of flavonoids and phenolics are influenced by the interaction between varieties and parts of plants. In fact, many medicinal plants contain large amount of antioxidants such as polyphenols. Previous studies have shown that some flavonoids components such as quercetin had anticancer activities and were able to inhibit cancer cell growth [24,25,26]. Gallic acid was reported as a free radical scavenger and as an inducer of differentiation and apoptosis in leukemia, lung cancer, and colon adenocarcinoma cell lines, as well as in normal lymphocyte cells [27,28]. It has been postulated that GA plays an important role in the prevention of malignant transformation and cancer development same as quercetin. Hence, the results of this research showed that flavonoids are important components of this plant, and some of its pharmacological effects could be attributed to the presence of these valuable constituents. Further work is required to establish if quercetin or any other flavonoids have any role in the prevention of this cancerous growth and development. 

### 2.2. Radical scavenging activity

#### 2.2.1. 1,1-Diphenyl-2-picryl-hydrazyl (DPPH) assay

**Figure 1:**
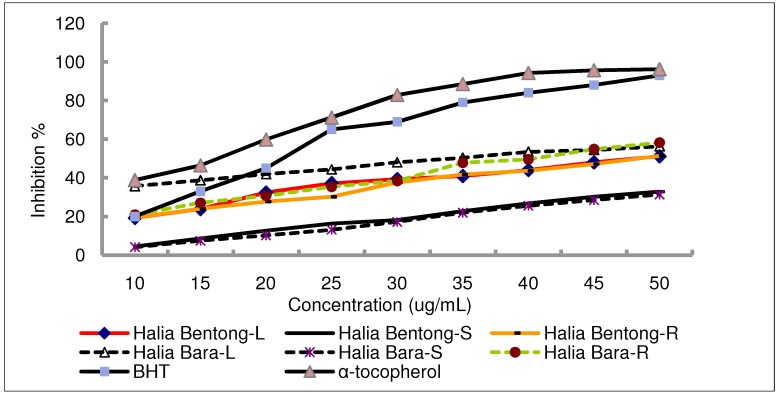
DPPH radical scavenging activity of the methanolic extracts in different parts of two varieties of *Zingiber officinale *compared with positive controls, BHT and α-tocopherol. L, S and R, respectively are: Leaves, Stems and Rhizomes of ginger.

It was observed that methanolic extracts of the leaves and rhizomes had higher activity than that of the stems (Table 2). At a concentration of 40 ug/mL, the scavenging activity of the methanol extract of Halia Bara leaves reached 50.35%, while at the same concentration, that of the stem was 27.38% (Figure 1). Similarly, using the same concentration for Halia Bentong, the scavenging activity of the methanol extract of the leaves and rhizome yielded 42.3 ug/mL, while that of the stem was 27.56%. The effect of antioxidants on DPPH is due to their hydrogen donating ability [29,30,31]. In this study, results showed that DPPH radical scavenging abilities of the extracts of plant parts were less than those of butylated hydroxyl toluene (BHT) (83.7%) and α-tocopherol (92.3%) at 40 ug/mL.

**Table 2:** DPPH scavenging activities of the methanolic extracts in different parts of two varieties of *Zingiber officinale. *BHT and α-tocopherol were used as positive controls.

Variety	Extract source	Inhibition %^a^
**Halia Bentong**	Leave	51.12 ± 1.65
	Stem	32.85 ± 0.57
	Rhizome	51.41±0.51
**Halia Bara**	Leave	56.36 ± 0.97
	Stem	31.45 ± 1.49
	Rhizome	58.22 ± 1.19
**Controls**	BHT	96.21 ± 0.24
	α-tocopherol	89.57 ± 1.12

All analyses were the mean of triplicate measurements ± standard deviation; a: Results expressed in percent of free radical inhibition.

This study showed that ginger methanolic extracts have good free radical scavenging ability and can be used as a radical inhibitor or scavenger, acting possibly as a primary antioxidant. The antioxidant activities of the leaves (51–56%) were also similar with the young rhizomes (51–58%), and the leaves could be served as food in the same way. Based on the results obtained, it is possible that several compounds of different polarities may contribute to the antioxidative properties of ginger leaves, stems, and rhizome extracts. Kikuzaki reported that methanolic extracts may include phenolic and hydrox-phenolic compounds with acid, alcohol, sugar or glycoside [12]. Part of the antioxidative activity may be due to these components or flavonoids. In addition, antioxidative activities observed in ginger varieties could be the synergistic effect of more than two compounds that may be present in the plant. 

### 2.3. Reducing ability

#### 2.3.1. Ferric Reducing Antioxidant Potential (FRAP)

The reducing ability of different parts of ginger extracts was in the range of 368.2–767.2 μm of Fe (II)/g dry weight (Table 3). In the leaves, rhizomes and stems, the antioxidant potentials in two varieties of *Zingiber officinale* were estimated from their ability to reduce 2,4,6-tripyridyl-*s*-triazine (TPTZ)-Fe (III) complex to TPTZ-Fe (II).

**Table 3:** Total antioxidant (FRAP) activity in different part of two varieties of *Zingiber officinale*. BHT, α-tocopherol and Vitamin C were used as positive controls.

Variety	Extract source	FRAP^a^
**Halia Bentong**	Leave	537.94 ± 37.3
	Stem	376.94 ± 50.97
	Rhizome	680.68 ± 18.38
**Halia Bara**	Leave	579.6 ± 61.94
	Stem	368.27 ± 23.43
	Rhizome	767.2 ± 41.53
**Controls**	BHT	74.31 ± 11.21
	α-tocopherol	953.0 ± 23.41
	Vitamin C	3107.28 ± 42.31

All analyses were the mean of triplicate measurements ± standard deviation; a: Results expressed in μmol Fe (II)/g dry weight.

The FRAP values for the methanolic extracts of the leaves, rhizomes and stems of in both varieties were significantly lower than those of ascorbic acid (vitamin C) and α-tocopherol, but higher than that of BHT. The ferric reducing ability (FRAP assay) is widely used in the evaluation of the antioxidant component in dietary polyphenols [20]. Antioxidant activity is found to be linearly proporational with phenolic contents. Oktay *et al.* reported a strong positive relationship between total phenolic contents and antioxidant activity, which appears to be the trend in many plant species [32]. 

## 3. Experimental

### 3.1. Plant material and maintenance

Two varieties of *Zingiber officinale *Roscoe (Halia Bentong and Halia Bara) seed rhizomes were germinated for two weeks in small pots and then transferred to 15 × 18 cm white polyethylene bags containing soilless mixture of burnt rice husk and coco peat at a ratio of 1:1. The plants were grown under glasshouse conditions at the glasshouse complex of University Putra Malaysia (UPM) where daily irradiance was approximately 790 umol m^-2 ^s^-1^. The plants were harvested after 16 weeks, with the leaves, stems, and rhizomes separated. Once dried, they were all kept at -80 ºC for future analysis. 

### 3.2. Extract preparation

Leaves, stems and rhizomes were freeze dried to constant weights prior to being used in the extraction. For antioxidant analysis, the leaves, stems, and rhizomes were powdered and 1 gram of the powder was extracted continuously with methanol (50 mL). The solution was then swirled for 1 h at room temperature using an orbital shaker. Extracts were then filtered under suction and stored at -20 ºC for further use.

### 3.3. Determination of total phenolic content

The total phenolic content was determined using Folin–Ciocalteu reagents with analytical grade gallic acid as the standard. 1 mL of extract or standard solution (0–500 mg/L) was added to deionized water (10 mL) and Folin–Ciocalteu phenol reagents (1.0 mL). After 5 minutes, 20% sodium carbonate (2.0 mL) was added to the mixture. After being kept in total darkness for 1 h, the absorbance was measured at 750 nm using a spectrophotometer (U-2001, Hitachi Instruments Inc., Tokyo, Japan). Amounts of TP were calculated using gallic acid calibration curve. The results were expressed as gallic acid equivalents (GAE) g/g of dry plant matter [33]. 

### 3.4. Determination of total flavonoid

A modified method [34] was used for this purpose/objective/part: diluted solution (1 mL) containing flavonoids, 5% (w/w) NaNO_2_ (0.7 mL) and 30% (v/v) ethanol (10 mL) were mixed for 5 min, and then 10% AlCl_3_ (w/w, 0.7 mL) was added and mixed altogether. Six minutes later, 1 mol/L NaOH (5 mL) was added. The solution was then diluted to 25 mL with 30% (v/v) ethanol. After standing for 10 min, the absorbance of the solution was measured at 430 nm with a spectrophotometer. A standard curve was plotted using quercetin as a standard. Different concentrations of quercetin were prepared in 80% ethanol and their absorbance was read at 430 nm using a spectrophotometer (U-2001, Hitachi Instruments Inc., Tokyo, Japan). The results were expressed in mg quercetin/g dry weight by comparison with the quercetin standard curve, which was made under the same condition. 

### 3.5. Determination of antioxidant activities

#### 3.5.1. DPPH radical scavenging assay

1,1-Diphenyl-2-picryl-hydrazyl (DPPH) was purchased from Sigma–Aldrich (USA). Butylated hydroxytoluene (BHT) and α-tocopherol were purchased from Merck (India). In order to determine the radical scavenging ability, the method reported by Mensor *et al.* [35], was used. Briefly, 0.3 mM alcohol solution of DPPH (1 mL) was added to samples (2.5 mL) containing different concentrations originating from different parts of ginger varieties’ extracts. The samples were first kept in a dark place at room temperature and their absorbance was read at 518 nm after 30 min. The antiradical activity (AA) was determined using the following formula: 

AA% = 100− ((Abs:sample _ Abs:empty sample)* 100)/ Abs:control

Blank samples contained 1 mL ethanol + 2.5 mL from various concentrations of ginger extract; control sample containing 1 mL of 0.3 mM DPPH + 2.5 mL ethanol. The optic density of the samples, the control and the empty samples were measured in comparison with ethanol. One synthetic antioxidant, BHT (butylhydroxytoluene) and α-tocopherol, were used as positive controls.

#### 3.5.2. Reducing ability (FRAP assay)

The determination of the total antioxidant activity using FRAP assay in the extract followed after a modified method reported by Benzie and Strain [36]. The stock solution included 300 mM acetate buffer (3.1 g C_2_H_3_NaO_2_·3H_2_O and 16 mL C_2_H_4_O_2_) at pH 3.6, 10 mM TPTZ (2,4,6-tripyridyl-*s*-triazine) solution in 40 mM HCl, and 20 mM FeCl_3_·6H_2_O solution in distilled water. Then acetate buffer (25 mL) and TPTZ (2.5 mL) were mixed together with FeCl_3_·6H_2_O (2.5 mL). The temperature of the solution was raised to 37 ºC before it was used. Plant extracts (150 μL) were allowed to react with the FRAP solution (2.85 mL) for 30 min under dark conditions. The absorbance was measured at 593 nm. The standard curve was linear between 200 and 1,000 μM FeSO_4_. Results were expressed in μM Fe (II)/g dry mass and compared with those of standards for BHT, ascorbic acid, and α-tocopherol.

### 3.6. Statistical analysis

The experimental results were expressed as mean ± standard deviation of three replicates. Where applicable, the data were subjected to one-way analysis of variance (ANOVA) and the differences between samples were determined by Duncan’s Multiple Range test using the Statistical Analysis System (SAS, 1999) programme. P-value of <0.05 was regarded as significant.

## 4. Conclusions

The results of this study indicated that *Zingiber officinale * has a high antioxidant activity and between the Malaysian varieties, Halia Bara possesses good medicinal potential. A positive relationship between antioxidant activities and total phenolic contents was also observed. The high level of total phenolic and flavonoid in Halia Bara variety indicated high antioxidant activities. This relationship was also reported in previous studies on other plants [37,38]. Further work is required to establish the components in phenolics and flavonoids that may have contributed to the high antioxidant activities so far observed.
